# Bacterial Efflux Pump Inhibitors Reduce Antibiotic Resistance

**DOI:** 10.3390/pharmaceutics16020170

**Published:** 2024-01-25

**Authors:** Lan Zhang, Xiaoyuan Tian, Lei Sun, Kun Mi, Ru Wang, Fengying Gong, Lingli Huang

**Affiliations:** 1 College of Veterinary Medicine, Huazhong Agricultural University, Wuhan 430070, China; zl-0118@webmail.hzau.edu.cn (L.Z.); shitxy@webmail.hzau.edu.cn (X.T.); sunlei23@webmail.hazu.edu.cn (L.S.); mikun@webmail.hzau.edu.cn (K.M.); w1665790632@webmail.hzau.edu.cn (R.W.); gongfengying@webmail.hzau.edu.cn (F.G.); 2 National Reference Laboratory of Veterinary Drug Residues, Huazhong Agricultural University, Wuhan 430070, China; 3 MAO Key Laboratory for Detection of Veterinary Drug Residues, Huazhong Agricultural University, Wuhan 430070, China; 4 MOA Laboratory for Risk Assessment of Quality and Safety of Livestock and Poultry Products, Huazhong Agricultural University, Wuhan 430070, China

**Keywords:** efflux pump inhibitor, drug resistance, biofilm, virulence, bacterial persister cells

## Abstract

Bacterial resistance is a growing problem worldwide, and the number of deaths due to drug resistance is increasing every year. We must pay great attention to bacterial resistance. Otherwise, we may go back to the pre-antibiotic era and have no drugs on which to rely. Bacterial resistance is the result of several causes, with efflux mechanisms widely recognised as a significant factor in the development of resistance to a variety of chemotherapeutic and antimicrobial medications. Efflux pump inhibitors, small molecules capable of restoring the effectiveness of existing antibiotics, are considered potential solutions to antibiotic resistance and have been an active area of research in recent years. This article provides a review of the efflux mechanisms of common clinical pathogenic bacteria and their efflux pump inhibitors and describes the effects of efflux pump inhibitors on biofilm formation, bacterial virulence, the formation of bacterial persister cells, the transfer of drug resistance among bacteria, and mismatch repair. Numerous efforts have been made in the past 20 years to find novel efflux pump inhibitors which are known to increase the effectiveness of medicines against multidrug-resistant strains. Therefore, the application of efflux pump inhibitors has excellent potential to address and reduce bacterial resistance.

## 1. Introduction

Bacterial infections seriously threaten food safety and public health, causing substantial economic losses and increasing the risk of zoonosis [1]. These infections can also lead to genomic instability of host cells and eventually to cancer [2]. As a result, bacterial infections remain a major challenge for modern medicine. Due to a lack of cross-protection from vaccines, the primary treatment for bacterial infections is still antibiotics. These drugs are used extensively in the medical, agricultural, and industrial fields and have reduced the severity and mortality of various infectious diseases caused by bacteria. However, their irrational clinical use has decreased drug efficacy and caused serious drug resistance problems.

According to survey data, each year, there are about 700,000 deaths worldwide caused by bacterial resistance [3]. If not controlled, this number will reach about 10 million by 2050. Moreover, bacterial resistance is expected to reduce the global gross domestic product by 2–3.5%. Global losses and consumption in antibiotic-resistance–related industries will reach USD 105 billion [4,5]. The World Health Organization first published a global priority list of antibiotic-resistant microorganisms in the beginning of 2017. Among them, *Pseudomonas aeruginosa*, *Enterobacteriaceae*, *Acinetobacter baumannii*, *Enterococcus faecium*, *Staphylococcus aureus*, and *Campylobacter* spp. constitute the main causes of life-threatening nosocomial infections throughout the world and are recognised as key, high-priority pathogens [6]. The antimicrobial resistance of pathogenic microorganisms is becoming increasingly serious, and the control of drug-resistant bacteria has become an urgent public health problem.

Over the past two decades, a variety of therapeutic treatments have been used to slow the development of antibiotic resistance, including (i) new drug development, (ii) multiple antibiotic combinations, (iii) phage therapy, (iv) antimicrobial peptide therapy, and (v) antibiotic–adjuvant combination approaches. However, new drug development is costly, has numerous difficulties in preparation, and the products may present structural instability. If we cannot address the growing problem of bacterial resistance, we may return to the pre-antibiotic era with no drugs to rely on.

Inhibitors have been widely used in recent years to delay bacterial resistance due to their high safety, low cost, and high efficiency, and they have great developmental value and application potential. The selection of inhibitors requires understanding the specific mechanisms of bacterial resistance, especially efflux pumps, which are widely expressed in Gram-positive and Gram-negative bacteria and mycobacteria. The overexpression of efflux pumps increases bacterial resistance to antibiotics, allowing them to survive at the injection site. Therefore, an essential way to inhibit drug-resistant bacteria is to search for and discover efflux pump inhibitors.

## 2. Mechanisms of Bacterial Resistance

### 2.1. The Main Resistance Mechanisms of Common Clinical Pathogens

Antibiotic resistance is the result of a complicated process involving multiple mechanisms. Bacteria have many different complementary mechanisms through which they can resist the effects of antibiotics [7]. Our summary of the resistance mechanisms of common clinical pathogens found that there are four main mechanisms through which bacteria become resistant to antibiotics: (i) by altering cell permeability to prevent antibiotics from entering cells, (ii) by altering an antibiotic’s molecular target such that it can no longer interact with the antibiotic, (iii) antibiotic inactivation through enzymatic modification, and (iv) the expression of an efflux pump, which keeps the antibiotic out of the cell’s surroundings (Table 1). These elements can be inherent or acquired through several processes. Additionally, free movement to human carriers and resistance determinants on mobile genetic components like plasmids and transposons might result in the spread of resistance to multiple microbial genera.

In response to exposure to various antimicrobial drugs, these pathogens have the capacity to quickly acquire or develop resistance mechanisms. Common to these pathogenic bacterial resistance mechanisms is a range of efflux pumps that efficiently exclude or reduce the intracellular concentration of large amounts of antibiotics, resulting in a significant increase in pathogenic resistance. In several bacteria, the upregulation of efflux pumps induced by antibiotic treatment underpins the initial intermediate resistance phenotype, which can result in higher-resistance mutants. Thus, efflux pumps are a target for creating innovative adjuvant medicines, and it is necessary to understand their purpose and contribution to drug resistance [7,15].

### 2.2. Bacterial Efflux System

On bacterial cells, membranes contain proteins called efflux pumps that control the flow of toxic materials from the internal cellular environment to the exterior cellular environment. Bacterial efflux pumps are the quickest acting and most efficient resistance mechanism for bacteria in response to stress, and they are present in almost all bacteria [16]. Efflux pumps improve the viability of microorganisms in challenging conditions and increase their resistance to antibiotics and antimicrobial agents [17].

**Figure 1:**
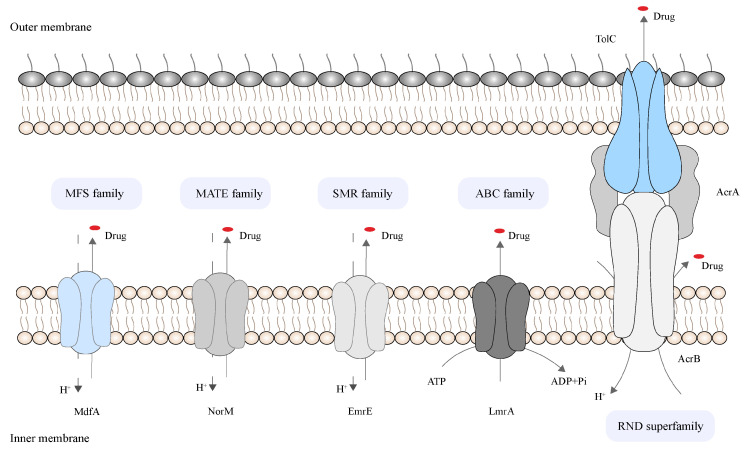
Representative structure diagram of five types of efflux pumps.

With the development of molecular microbiology, many bacterial efflux pumps have been identified, including in methicillin-resistant *Staphylococcus aureus* (MRSA), *Klebsiella pneumoniae*, *Streptococcus pneumoniae*, *Pseudomonas aeruginosa*, *Listeria monocytogenes*, *Acinetobacter baumannii*, *Enterococcus* spp., *Escherichia coli*, *Campylobacter jejuni*, and *Salmonella* spp. [18]. Six bacterial drug efflux pump families have been identified to be involved in the efflux pathway, including the ATP-binding cassette (ABC) family, the resistance-nodulation-cytosis (RND) superfamily, the small multidrug resistance (SMR) family, the multidrug and toxin extrusion (MATE) family, the major facilitator superfamily (MFS), and the proteobacterial antimicrobial complex efflux (PACE) family. The RND family is found only in Gram-negative bacteria. Among them, the five other families of secondary active transporters are driven by electrochemical energy captured by transmembrane ion gradients; the ATP-binding cassette (ABC) family uses ATP as a source of energy for transport (Figure 1) [19]. An efflux pump can excrete a variety of substrates, often antibiotics, thus leading to efflux-pump-expressing bacterial antibiotic resistance phenotypes. Efflux pumps can also excrete detergents, dyes, toxins, and waste metabolites [20,21]. Table 2 presents examples of the main relevant efflux pumps for common clinical bacteria.

## 3. The Role of Efflux Pump Inhibitors in Reversing Drug Resistance

### 3.1. The Status of Drug Resistance Reversal by Efflux Pump Inhibitors

Efflux pump inhibitors block efflux pumps through one or more processes, which can inactivate drug transport. To increase the action of antibiotics against bacteria expressing efflux pumps and restore the effectiveness of antibiotics that are no longer active against a pathogen, efflux pump inhibitors can be used as adjuvants with antibiotics [21]. Efflux pump inhibitors promote the intracellular accumulation of multiple antibiotics at the same time, making bacteria more susceptible to antimicrobial drugs. The effect of an inhibitor usually has two consequences: first, it will re-sensitise resistant microorganisms to currently used antibiotics. Second, it will lessen the opportunity to select resistant mutants [31]. Bacterial efflux pump inhibitors are available from a wide variety of sources, both natural and synthetic compounds (Table 3). Based on the global priority list of antibiotic-resistant microorganisms published by the World Health Organization, the discovery of efflux pump inhibitors for high-priority pathogens is urgent and significant.

#### 3.1.1. *Staphylococcus aureus* Efflux Pump Inhibitors

*Staphylococcus aureus* is a common zoonotic pathogen that causes tissue-limited suppurative infections, and poorly controlled infections may result in adverse outcomes such as extension of infection and bacteraemia. *S. aureus* is the most common Gram-positive bacterium in clinical infections. Antibiotic treatment is the first choice for *S. aureus* infections, but it is difficult to treat *S. aureus* infections because of its high resistance and because the disease recurs easily. Determining how to treat infections caused by drug-resistant *S. aureus* is a hotspot in current research. Efflux-mediated resistance has recently attracted more attention in *S. aureus* than other resistance mechanisms, and the inhibition of the activity of drug efflux pumps reduces the bacterial efflux of drugs and increases bacterial susceptibility to drugs. Thus, efflux pump inhibitors are important substances in combatting drug-resistant *S. aureus*.

**Figure 2:**
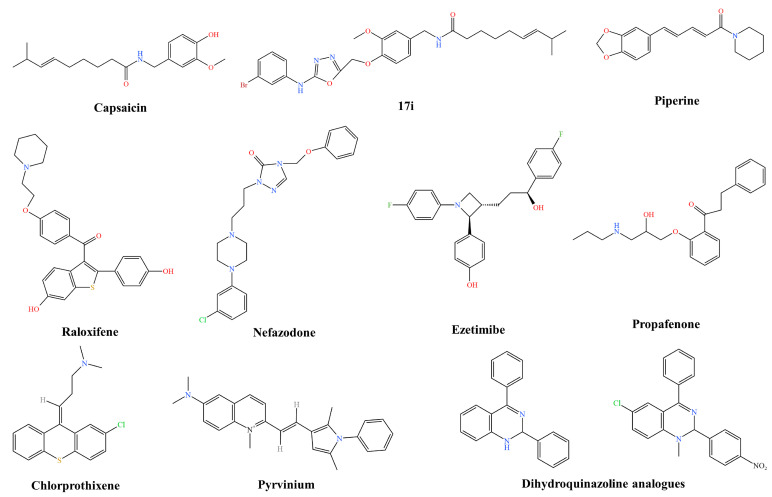
Inhibitor structural formula of *Staphylococcus aureus*.

Capsaicin (Figure 2), a novel NorA efflux pump inhibitor, not only increases the inherent susceptibility of *S. aureus* to ciprofloxacin but also remarkably reduces the emergence of ciprofloxacin-resistant mutant strains. The combination of ciprofloxacin and capsaicin has a bactericidal effect and enhances the post-antibiotic effect (PAE) of ciprofloxacin in a concentration-dependent manner [49]. Due to its poor water solubility, significant first-pass effect, and low oral bioavailability, capsaicin is currently used as a topical agent for a wide range of diseases, including sciatica, rheumatism, and lumbago [50]. The Food and Drug Administration (FDA) and the European Medicines Agency (EMA) approved a prescription drug containing capsaicin as the active ingredient in 2009 for the treatment of peripheral neuropathic pain in adults, either alone or in combination with other pain medications. Considering the potential of capsaicin to inhibit the NorA efflux pump, researchers prepared a range of capsaicin-based 1,3,4 oxadiazole conjugates via the single-site modification of the hydroxyl group of the vanilloid group of capsaicin and evaluated their potentiation of ciprofloxacin activity. Of all the compounds, 17i (Figure 2) showed robust activity, and the minimum effective concentration (MEC) of this inhibitor against SA1199B, a NorA-overexpressing strain, was 12.5 μg/mL, which is superior to that of its parent coupler, capsaicin (MEC 50 μg/mL). Compound 17i enhanced the activity of ciprofloxacin fourfold at an MEC of 12.5 μg/mL and reduced the mutation prevention concentration (MPC) of ciprofloxacin. In addition, 17i reduced EtBr efflux, confirming the inhibitory effect on NorA [51].

Piperine (Figure 2) is an important natural compound with a variety of health benefits. Despite its low absorption and bioavailability due to hydrophobicity, its ability to participate in herb–drug or herb–herb interactions also contributes to the pharmacokinetic and dynamic properties of other drugs/nutraceuticals [52]. The activity of ciprofloxacin against *S. aureus* is increased by piperine and its analogues, which also act as NorA inhibitors by lowering the minimum inhibitory concentration (MIC) of ciprofloxacin in *S. aureus* 1199B. Piperine also acts as an inhibitor of the *S. aureus* MdeA efflux pump, reducing the mupirocin MIC by 2–4-fold in *S. aureus* ATCC 29213 and MRSA isolates. The relative probability of selecting resistant mutants for the combination of mupirocin and piperine is significantly lower compared with mupirocin alone. Piperine is on the FDA’s list of “Generally Regarded as Safe” (GRAS) compounds. Therefore, there should be no toxicity issues with topical preparations of piperine and mupirocin, and piperine may prove to be useful for nasal debridement and skin infections [35].

Mahey N et al. screened a library of FDA-approved small-molecule drugs and determined six potential compounds using molecular docking which are chlorprothixene, ezetimibe, raloxifene, nefazodone, propafenone, and pyridinium (Figure 2). These compounds acted synergistically with ciprofloxacin and reduced its MIC to less than or equal to the clinical resistance breakpoint in overexpressed norA as well as in several clinical *S. aureus* strains. Furthermore, raloxifene, ezetimibe, and pyridinium showed synergistic effects with norfloxacin. In norA-overexpressing strains, the MIC was reduced to the clinical resistance breakpoint. Many of the early EPIs discovered had some toxicity concerns due to their off-target effects. In the current study, none of the working concentrations of EPIs interfered with membrane function or depleted the overall bacterial ATP pool, and the synergistic effect embodied by the drugs suggests that the EPIs of the drugs have a genuine similarity rather than being the results of any off-target effects [22].

Dihydroquinazoline, a complete class of heterocyclic compounds, has synergistic effects with norfloxacin and ethidium bromide. Of the seventeen dihydroquinazolines synthesised and screened for possible efflux pump inhibitor activity against *S. aureus* 1199B, eight proved to be effective inhibitors, significantly reducing the MICs of norfloxacin and ethidium bromide against *S. aureus* 1199B by up to 16-fold. Dihydroquinazoline analogues (Figure 2) also significantly reduce norA gene expression in the presence of subinhibitory concentrations of norfloxacin [53].

#### 3.1.2. *Klebsiella pneumoniae* Efflux Pump Inhibitors

*Klebsiella pneumoniae* is a Gram-negative opportunistic pathogen that can cause serious hospital-acquired infections such as sepsis, pneumonia, urinary tract infections, soft tissue infections, and surgical site infections [54]. The increasing mortality rate due to drug-resistant *K. pneumoniae* infections has caused global alarm. Drug efflux pumps are a critical mechanism of drug resistance in *K. pneumoniae*. They continuously pump out drugs that have infiltrated the bacteria, resulting in low enterobacterial drug concentrations that are insufficient to exert antibacterial effects. To address the severe problem of efflux-pump-mediated multidrug resistance, many efflux pump inhibitors have been identified and tested.

**Figure 3:**
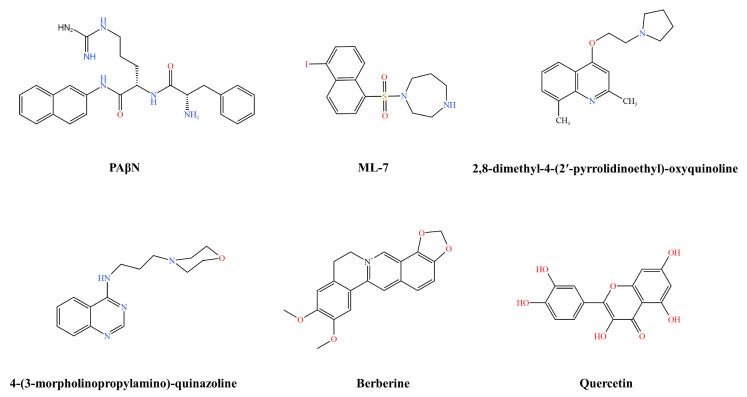
*Klebsiella pneumoniae* inhibitor structural formulae.

One of the best characterized efflux pump inhibitors for Gram-negative bacteria is β-naphthylamide (PAβN) (Figure 3). PAβN is a competitive EPI and is considered a broad-spectrum pump substrate. This compound significantly reduces the MICs of chloramphenicol, tetracycline, and quinolones against multidrug-resistant *K. pneumoniae*. Chloramphenicol accumulation assays revealed low levels of chloramphenicol accumulation in cells in the absence of an inhibitor. In contrast, the use of the inhibitor resulted in a 3–4-fold increase in intracellular drug concentration, and the addition of PAβN significantly inhibited the activity of the efflux mechanism [55]. 

Tigecycline is regarded as the final line of defence in treating severe infections caused by multidrug-resistant (MDR) Gram-negative bacteria, especially extensively drug-resistant (XDR) Enterobacteriaceae bacteria. Sun et al. conducted a preliminary screening of 2711 compounds in combination with tigecycline using clinical isolates of *K. pneumoniae*. The results indicated that ML-7 (Figure 3) and tigecycline had synergistic effects on 11 strains of clinically isolated *K. pneumoniae* and could reduce the MIC of tigecycline by 4–128-fold. These *K. pneumoniae* belonged to different sequence types, suggesting that the ML-7-induced reduction in the MIC of tigecycline may be a common pattern for isolates of different sequence types of *K. pneumoniae*. ML-7 could disrupt the function of the multidrug efflux pump and promote the accumulation of tigecycline in cells. The potential applicability of ML-7, an inhibitor of myosin light chain kinase (MLCK), in bacterial infections has not been reported to date, and studies on ML-7 are still in the in vitro phase, but its potential as a novel tigecycline adjuvant to reverse drug resistance should not be overlooked [56].

Quinoline and quinazoline derivatives have been reported to be useful chemical sensitisers in efflux-pump-overexpressing strains. Alkylaminoquinolines, alkoxyquinolines, and chloroquinolines are considered broad-spectrum efflux pump inhibitors that increase susceptibility to chloramphenicol, norfloxacin, and tetracycline-resistant *K. pneumoniae* [31]. 2,8-dimethyl-4-(2′-pyrrolidinoethyl)-oxyquinoline (Figure 3) reduced the MIC of chloramphenicol by 16-fold, and 4-(3-morpholinopropylamino)-quinazoline (Figure 3) reduced the MICs of chloramphenicol, nalidixic acid, and tigecycline by 32-, 32-, and 8-fold, respectively [57,58]. Quinazoline derivatives are also effective at increasing the susceptibility of *K. pneumoniae* to chloramphenicol and nalidixic acid [59].

Berberine (Figure 3) has antibacterial, antiviral, anti-yeast, antiparasitic, antifungal, and anti-*Candida albicans* activity and is considered an antibacterial agent in Chinese and Western medicine. Berberine has low oral absorption into the blood and large intestinal excretion [60]. Previous studies have shown that berberine can be extensively metabolised and that metabolites also contribute to its therapeutic effect [61]. In one study, combining berberine with ciprofloxacin reduced ciprofloxacin concentrations by one-half to three-fourths in *K. pneumoniae*. There was a synergistic effect in 18.18% of the isolates. In addition, combining ciprofloxacin with berberine resulted in bacterial stagnation in a time–kill assay. These findings suggest that berberine enhances bacterial susceptibility to ciprofloxacin [62]. Berberine is currently approved for marketing in China and is mostly used in tablets. Berberine hydrochloride tablets belong to the group of digestive and metabolic drugs used as antidiarrhoeal, intestinal anti-inflammatory, and anti-infective medications.

Quercetin is a flavonoid found in fruits and vegetables that is poorly absorbed, extensively metabolised, and rapidly eliminated. Its unique biological profile forms the basis for potential benefits to overall health and disease resistance, including anticancer, anti-inflammatory, antibacterial, antiviral, and antioxidant properties [63]. Many virtual screening and molecular docking-based investigations, as well as particular fluorescence and mass spectrometry-based techniques, have shown quercetin to be an active efflux pump inhibitor over the past 20 years. Pal et al. evaluated carbapenemase and the efflux pump inhibition potential of quercetin (Figure 3) using an enzyme inhibition assay and an efflux pump inhibition assay, respectively. Quercetin at 1/2 MIC (64 µg/mL) showed an approximately 1.7-fold inhibition of AcrB efflux activity in *K. pneumoniae* overexpressing carbapenemase, and its MIC was reduced when combined with meropenem [64]. Quercetin is currently in the clinical research phase, and clinical trials have been conducted to evaluate quercetin in prostate cancer (NCT01912820), COPD (NCT03989271), and COVID-19 infection (NCT04468139) [65].

#### 3.1.3. *Pseudomonas aeruginosa* Efflux Pump Inhibitors

*Pseudomonas aeruginosa* is a common conditionally pathogenic Gram-negative bacterium mainly found in intensive care units which can cause diseases such as intraoperative port infections and chronic respiratory infections [66]. The misuse of antibiotics in recent years has made *P. aeruginosa* widely resistant to cephalosporins, polymyxins, and sulphonamides, increasing the difficulty of clinical anti-infective treatment [67]. Efflux pumps are the material basis of their multi- and pan-drug resistance, mediating natural and acquired resistance. The discovery of efflux pump inhibitors is the key to solving the *P. aeruginosa* drug resistance problem.

**Figure 4:**
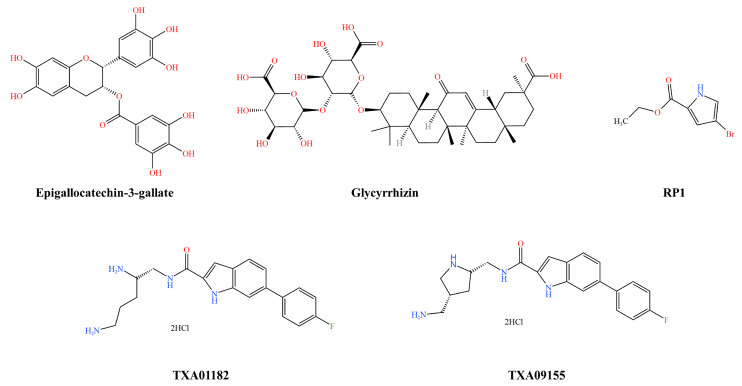
*Pseudomonas aeruginosa* inhibitor structural formulae.

Epigallocatechin-3-gallate (EGCG) (Figure 4) is a polyphenol compound extracted from green tea leaves that has antimicrobial activity and several health benefits. Subinhibitory concentrations of EGCG significantly enhanced antibiotic susceptibility, especially to chloramphenicol and tetracycline (≥4-fold). The enhanced antimicrobial activity and EGCG-mediated antibiotic susceptibility of EGCG in externally pumped (MexAB-OPrM) mutant strains compared to parental strains suggest a potent externally pumped inhibitory effect [42]. Like other phenolic compounds, EGCG has low bioavailability. Researchers have proposed that the relevant effects of EGCG in the body may not be caused by itself but rather by its metabolites, as it is metabolised by intestinal flora before it is absorbed into the bloodstream [68]. EGCG has promising applications with important protective effects against metabolic diseases, cerebral ischaemic injury, neurodegenerative diseases, tumours, and cardiovascular diseases. In addition, EGCG was the first botanical drug approved by the FDA to play a vital role in the treatment of genital warts as an anti-infective prescription [69].

Glycyrrhizin (GLY) (Figure 4) is a glycyrrhizic triterpenoid extracted from liquorice root (Glycyrrhiza glabra) which has a variety of properties and is used in the clinical management of chronic hepatitis. GLY is poorly absorbed in the human gastrointestinal tract [70], but GLY has been shown to increase the bioavailability of antibiotics, making them more effective at lower concentrations. The combination of ciprofloxacin and GLY with ciprofloxacin reduced its MIC. GLY altered the permeability of bacterial cell membranes and reduced bacterial survival. GLY significantly reduced the mRNA expression levels of the RND efflux pumps tested, and the fluorescence accumulation of EtBr was enhanced by the addition of the inhibitor, which suggests reduced efflux activity. In mouse treatment trials, GLY and ciprofloxacin treatment significantly reduced clinical scores, plate counts, and Myeloperoxidase (MPO) compared to ciprofloxacin [71].

Ethyl 4-bromopyrrole-2-carboxylate (RP1) (Figure 4) was isolated from the soil bacterium Streptomyces IMTB 2501. RP1 acted synergistically with an antibiotic by reducing its MIC in strains overexpressing the prototypical RND transporter protein (MexAB-OprM). The antibiotic-RP1 combination prolonged the after-effects of the antibiotic and reduced the mutation-preventive concentration of the antibiotic [72]. 

Yuan et al. evaluated the potential of heterocyclic formamide as an efflux pump inhibitor for *P. aeruginosa*. Among several types of thickened heterocyclic formamides, the aryl indole formamide compound TXA01182 (Figure 4) at 6.25 µg/mL showed an eightfold enhancement of levofloxacin with synergistic effects on several multidrug-resistant *P. aeruginosa* clinical isolates, reducing the frequency of antimicrobial agent resistance and enhancing the killing kinetics of levofloxacin. The authors also reported synergistic activity with other antibacterial classes (fluoroquinolones, sulphonamides, and tetracyclines) against *P. aeruginosa*, resulting in a 4–32-fold reduction in the MIC [73]. Later, Zhang et al. improved the efflux pump inhibitor activity and overall pharmacological properties of TXA01182 and discovered TXA09155 (Figure 4). This novel efflux pump inhibitor shows robust potentiation when combined with multiple antibiotics. In both wild-type and multidrug-resistant clinical isolates of *P. aeruginosa*, it increases the effectiveness of numerous antimicrobial drugs with efflux capacity while contributing little to membrane disruption and the prevention of resistance flare-ups. TXA09155 at 6.25 µg/mL showed an 8–256-fold enhancement of levofloxacin, doxycycline, moxifloxacin, chloramphenicol, cefpirome, cotrimoxazole, and minocycline which reduced the prevalence of levofloxacin resistance and improved the killing kinetics of moxifloxacin [74].

#### 3.1.4. *Escherichia coli* Efflux Pump Inhibitors

Foodborne pathogens are an essential cause of food poisoning and other related diseases and pose an important threat to food safety. *Escherichia coli*, as an extremely widely distributed pathogen, is one of the main factors causing foodborne diseases, posing a great threat to human health, and controlling the contamination of food with *E. coli* is particularly important. Of bacteria, *E. coli* has the most types of efflux pumps, and drug-resistant strains often carry antimicrobial drug-resistance genes against aminoglycosides, quinolones, chloramphenicol, and sulphonamides, among others. A bacterium can have multiple resistance mechanisms and show characteristics of multidrug resistance. We can solve the problem of *E. coli* drug resistance by discovering novel efflux pump inhibitors or structural modifications to known antibiotics.

**Figure 5:**
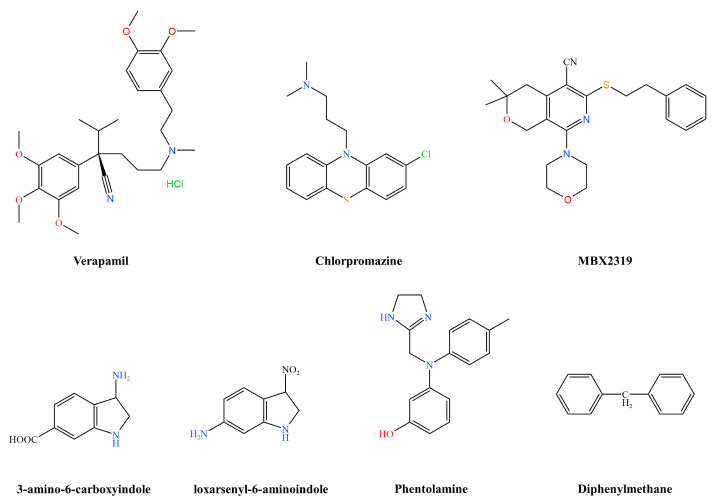
*Escherichia coli* inhibitor structural formulae.

MFS efflux system inhibitors work by reducing the level of resistance of *E. coli* to different fluoroquinolones. Verapamil (Figure 5) reduced the MICs of norfloxacin, levofloxacin, and gatifloxacin by 2–6-fold, and chlorpromazine (Figure 5) had the same effect on norfloxacin, levofloxacin, and gatifloxacin [75]. Currently, Verapamil is approved by the FDA for marketing as a prescription drug in the form of tablets, capsules, injections, and solutions for the treatment of cardiovascular diseases. Similarly, chlorpromazine was approved by the FDA for marketing as a prescription drug in tablet form for the treatment of neurological disorders. MBX2319 (Figure 5) is a new pyrano-pyridine that acts as a potent efflux pump inhibitor of the RND efflux system in *Enterobacteriaceae* bacteria. MBX2319 reduced the MICs of ciprofloxacin, levofloxacin, and piperacillin against *E. coli* strains by two, four, and eight times, respectively, but shows no activity against strains lacking AcrAB-TolC [44]. Zeng et al. created synthetic indole derivatives based on the TolC structure. They reported that 3-amino-6-carboxyindole and loxarsenyl-6-aminoindole (Figure 5) had synergistic antibacterial activity with ciprofloxacin, tetracycline, chloramphenicol, and erythromycin against *E. coli* strains overexpressing the AcrAB pump, resulting in a 2–64-fold reduction in the MIC [76]. 

Cui et al. combined phentolamine (Figure 5) with the macrolide antibiotics azithromycin, clarithromycin, and erythromycin and found a synergistic effect on *E. coli* test strains, as indicated by a fractional concentration inhibition index (FICI) of 0.375–0.5. The kinetic time–kill assay revealed that the bactericidal activity of all three macrolides against the strains increased in the presence of phentolamine. The combination of antibiotics and phentolamine reduced bacterial density to above 2log_10_ CFU/mL, again indicating synergy. Phentolamine is a reversible, non-selective alpha-adrenergic antagonist that has been approved for marketing by the FDA. The compound has been successfully used in the treatment of cocaine-induced cardiovascular complications to counteract serious peripheral vasoconstriction and pain caused by the infusion of vasopressors. Numerous investigations and clinical data confirm its safety profile, and its reversal of resistance to macrolide antibiotics highlights its potential as an efflux pump inhibitor [77].

Seaweed is rich in polysaccharides, pigments, fatty acids, polyphenols, and peptides which have antioxidant, antibacterial, and anti-inflammatory effects. The seaweed compound diphenylmethane (DPM) (Figure 5) could regulate the activity of macrolides and fluoroquinolones against drug-resistant *E. coli* and reduce the half-maximal inhibitory concentration (IC_50_) values of clarithromycin, ciprofloxacin, and erythromycin by twofold. According to a time-killing experiment, DPM and erythromycin together showed a more substantial inhibitory effect against drug-resistant *E. coli*. The study of ethidium bromide efflux showed that the addition of DPM significantly reduced dye efflux, indicating that it interferes with the efflux pump [78]. 

#### 3.1.5. *Campylobacter jejuni* Efflux Pump Inhibitor

*Campylobacter jejuni* is a Gram-negative and spiral-shaped microaerobic bacterium that is a major cause of foodborne infections in humans in both developed and developing countries [79]. Fluoroquinolones and macrolides are the most commonly used antibiotics to treat *Campylobacter* infections, but the increasing resistance of these bacteria to clinically essential antibiotics is a severe public health problem. The multidrug efflux pump CmeABC stands out among the factors that influence antibiotic resistance in *C. jejuni*. Recent studies have also identified various compounds with significant inhibitory effects on CmeABC.

**Figure 6:**
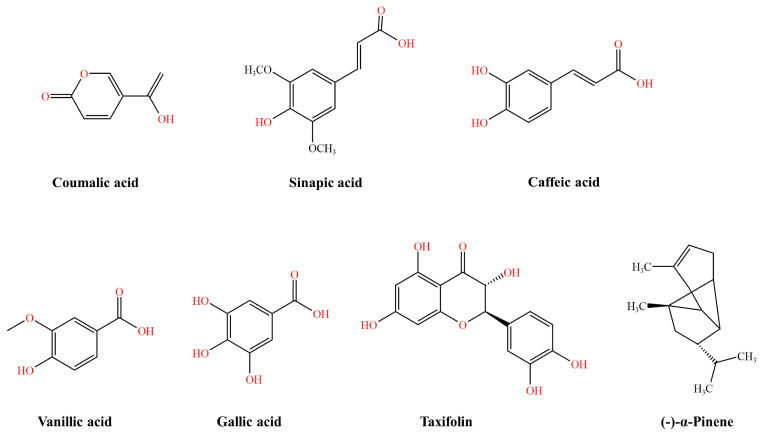
*Campylobacter jejuni* inhibitor structural formulae.

Phenolic compounds are secondary metabolites of plants; some of them have antibacterial activities against various pathogenic bacteria. Polyphenols are not necessarily the most active in the body, possibly because they are poorly absorbed from the gut, highly metabolised, or rapidly eliminated [80]. Once absorbed through the intestinal barrier, polyphenols are extensively metabolised in tissues [81]. However, polyphenols can affect the bioavailability of many therapeutic drugs by influencing the activity of various enzymes involved in their own metabolism [80], thereby enhancing antibiotic efficacy and antimicrobial action. Oh et al. observed the synergistic antibacterial activity of six phenolic compounds, including five phenolic acids (coumalic acid, sinapic acid, vanillic acid, galluc acid, and caffeic acid) and one flavonoid (taxifolin) (Figure 6). Specifically, 8 μg/mL of each phenolic component decreased the MICs of ciprofloxacin and erythromycin in five distinct *C. jejuni* strains, including three humans and two avian isolates, by roughly 4–32-fold [82]. Caffeic acid has been approved for use in China in the form of tablets as an anti-haemorrhagic agent. Essential oil constituents like the monoterpene (−)-pinene (Figure 6) can stop Gram-negative efflux pumps. (−)-α-Pinene can inhibit the CmeABC efflux pump, thereby increasing the susceptibility of *C. jejuni* to triclosan, ciprofloxacin, and erythromycin [83]. α-Pinene is the main active ingredient in eucalyptol, limonene, and pinene enteric capsules which have been approved for use in China as a respiratory medicine. Martinez et al. investigated the effect of Phe-Arg β-naphthylamide dihydrochloride (MC-207,110) on the function and resistance of CmeABC. The efflux pump inhibitor effectively decreased both inherent and acquired resistance to erythromycin in *C. jejuni* (MIC decreased 64–128-fold), with a fluoroquinolone MIC 2–4–fold lower [84].

### 3.2. Screening Methods and Key Technologies of Efflux Pump Inhibitors

A suitable screening method is a prerequisite and an essential guarantee for screening drugs. At present, new drug screening technologies are developing very rapidly. The various screening methods complement and validate each other, providing crucial technical support to screen potentially valuable inhibitors.

Virtual screening is a database search method based on molecular docking technology. It is mainly applied to screen small-molecule inhibitors against enzymes, proteins, and other known three-dimensional-structure receptors [85]. After the docking calculation has been completed for all molecules in the library, the best molecule that binds to the target can be identified by scoring the docked molecules according to their interaction and binding energy [86]. The goals of virtual screening are to discover target compounds from an extensive library of small molecules, to reduce the number of compounds screened in physical experiments, to shorten the study period, and to save research costs. 

The checkerboard test, which identifies synergistic combinations of antimicrobial agents, was used to screen for potential efflux pump inhibitors. Successive twofold dilutions of antibiotics and tested compounds were made in bacterial culture plates with different combinations of concentrations [32]. The fractional inhibitory concentration (FIC) index was used to determine the combined bacterial inhibitory effect. An FIC index ≤ 0.5 is a synergistic effect; 0.5 < FIC index ≤ 1 is additive effect; 1 < FIC index ≤ 4 is irrelevant; and an FIC index > 4 is antagonistic effect.

$$\mathrm{FIC}\;\mathrm{index}=\frac{\mathrm{MIC}\;\mathrm{of}\;A\;\mathrm{in}\;\mathrm{combination}\;\mathrm{with}\;B}{\mathrm{MIC}\;\mathrm{of}\;A\;\mathrm{alone}}+\frac{\mathrm{MIC}\;\mathrm{of}\;B\;\mathrm{in}\;\mathrm{combination}\;\mathrm{with}\;A}{\mathrm{MIC}\;\mathrm{of}\;B\;\mathrm{alone}}$$

A general approach to testing the validity of efflux pump inhibitors is to measure the growth inhibition of antibiotics in different bacterial strains in the absence and presence of efflux pump inhibitors. In experiments, wild-type, knockout, and efflux-pump-overexpression strains are often used. Greater synergy in efflux-pump-overexpressing strains compared with wild-type strains and the lack of growth inhibition in knockout strains are indicators of an efflux pump inhibitor. In this way, researchers can examine the intrinsic antimicrobial activity of potential inhibitors to demonstrate that the reduction in an antibiotic MIC is not the result of other mechanisms of antimicrobial activity [6]. 

In addition, a fluorescence real-time efflux analysis is an equally important tool for determining the efficacy and specificity of efflux pump inhibitors [32]. The most commonly used compound in efflux analysis is ethidium bromide (EtBr), which fluoresces only when embedded in DNA within the cell. In this test, adding an energy source, such as glucose, to the cells causes a fluorescent dye to extrude. If the efflux is reduced, fluorescence is retained over time. Therefore, an increase in fluorescence with the addition of efflux pump inhibitors is an important indicator of presumed activity. However, severe optical interferences were observed when assessing the efflux pump inhibitory activity of plant extracts and some purified compounds by fluorescence detection due to matrix burst effects in crude extracts and even in some pure compounds. In order to avoid the problem of optical matrix interference, Brown et al. developed a liquid chromatography–mass spectrometry (LC-MS) method for the determination of the concentration of ethidium bromide in fermentation broth filtrates. The LC-MS method produces data with a concentration–response relationship opposite to that produced by fluorescence measurements, i.e., as the concentration of the inhibitor is increased, the inhibitor reduces the exocytosis of EtBr by trapping it in the bacterial cells, and the EtBr concentration in the waste filtrate (measured by LC-MS) decreases [87]. However, EtBr is a DNA insertion agent, and there are concerns about its mutagenicity. One study optimised the method and established a novel DiOC_3_ 96-well real-time exclusion assay suitable for high-throughput efflux pump inhibitor screening using *S. aureus*. This approach does not use a potentially dangerous DNA insertion agent [88]. The use of virtual screening and physical experiments to complement and validate each other provides essential technical support to screen potentially valuable inhibitors.

## 4. The Gain Effect of Efflux Pump Inhibitors

### 4.1. Efflux Pump Inhibitors Inhibit Biofilm Formation

Cells are frequently embedded in self-produced extracellular polymer matrices that are irrevocably connected to the matrix or interface or each other, forming biofilms, which are groups of microorganisms that have been confined. They are usually found in industrial, medicinal, and natural environments on moist surfaces [89]. Numerous environmental cues, such as host-derived signals, nutritional and metabolic cues, mechanical signals, population signals (quorum sensing (QS)), and subinhibitory antimicrobial concentrations, all favour the production of biofilms [90]. Biofilms protect bacterial cells from immune defences and antibiotics, making bacteria highly resistant to antimicrobial drugs.

**Figure 7:**
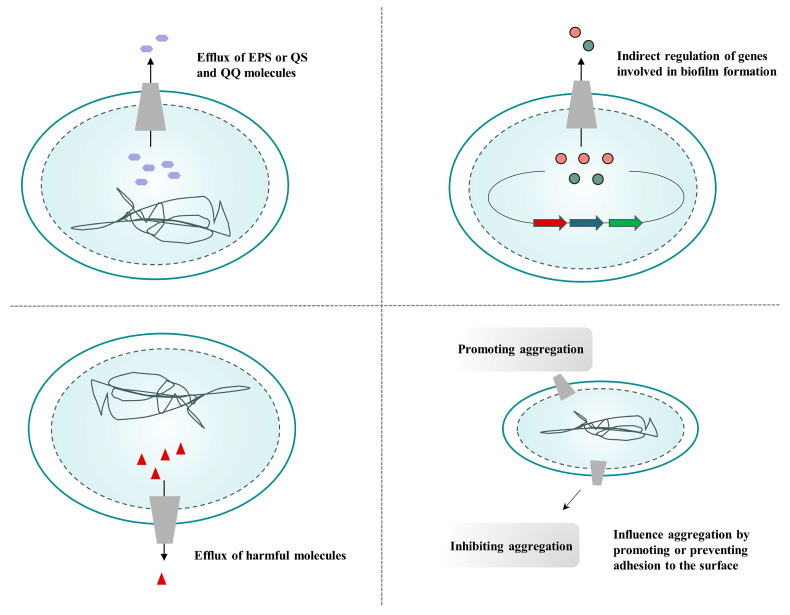
Four different potential roles of efflux pumps in biofilm formation. Adapted from Alav et al. [91].

At least four functions for efflux pumps in the growth of biofilms are possible (Figure 7): (i) biofilm matrix production and QS regulation are both aided by extracellular polymeric substances (EPSs), QS efflux, and population quenching (QQ) molecules; (ii) the indirect control of biofilm-formation-related genes; (iii) the efflux of harmful substances, including antibiotics and metabolic byproducts; and (iv) encouraging or inhibiting adherence to surfaces and other cells to affect aggregation [91]. Antibiotic resistance in biofilms has been linked to the putative multidrug resistance pump YhcQ in *E. coli*. MexAB-OprM and MexCD-OprJ efflux pumps have been implicated in resistance mechanisms specific to biofilms in *P. aeruginosa* [92]. Efflux pumps are actively involved in biofilm formation, so interfering with their function may impair biofilm formation.

Kvist et al. found that efflux pump and drug transporter protein genes are highly upregulated during biofilm growth and demonstrated the value of efflux pump inhibitors as potential anti-biofilm drugs. The effectiveness of three known efflux pump inhibitors, PAβN, azoxystrobin, and 1-(1-naphthyl methyl) piperazine (NMP), was tested against *S. aureus*, *K. pneumoniae*, *P. aeruginosa*, and *E. coli*. In almost all strains, the inhibitors significantly reduced biofilm formation [92]. Singh et al. demonstrated that boeravinone B inhibits efflux-pump-mediated fluoroquinolone efflux as well as the formation of an *S. aureus* biofilm. In the presence of boeravinone B (6.25–25 M), there was a concentration-dependent reduction in the minimum biofilm inhibitor concentration (MBIC) of ciprofloxacin against both bacteria. The staining showed a significant decrease in cell viability in the biofilm after the addition of the inhibitor. The combination of ciprofloxacin and the inhibitor showed a better biofilm inhibitory effect [93]. Vitamin C (VC) is a potential antioxidant. Prior research showed that VC may effectively kill Gram-positive organisms by causing oxidative stress and could prevent *E. coli* from forming biofilms. Xuet al. then demonstrated that VC could effectively treat infections caused by carbapenem-resistant hypervirulent *Klebsiella pneumoniae*. the subminimum inhibitory concentration (sub-MIC) of VC functioned as an efflux pump inhibitor, obstructing the passage of EPS to the bacterial cell surface and thus impeding the formation of biofilms. Both confocal laser scanning microscopy and scanning electron microscopy revealed that at lower VC concentrations, the biofilm structure was fragmented and began to disintegrate [94]. 

This suggests to us that due to the intrinsic link between efflux pumps and biofilms, some agents are potent biofilm inhibitors due to their excellent efflux pump inhibition. Efflux pump inhibitors have great potential as anti-biofilm drugs.

### 4.2. Efflux Pump Inhibitors Reduce Bacterial Virulence

The issue of antibiotic resistance has encouraged the creation of novel therapies, one of which is the discovery of substances that diminish the pathogenic potential of toxic microbes. These compounds, which can be used alone or in combination with antibiotics, can help treat bacterial infections by impairing the ability of bacterial pathogens to proliferate in infected patients [95]. The potential interaction between the antibiotic resistance, virulence, infectivity, and pathogenicity of pathogens mediated by the efflux system emphasises the possible use of efflux pump inhibitors as antivirulence and antiresistance substances.

**Figure 8:**
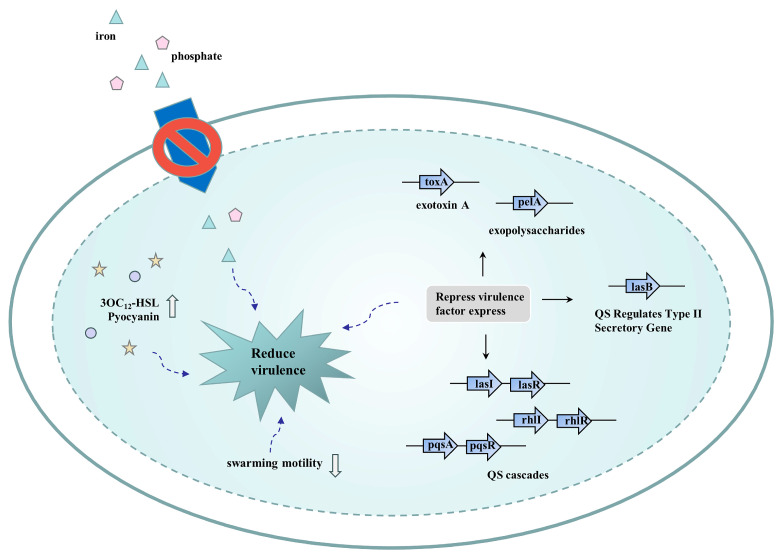
Possible strategies to reduce bacterial virulence.

Under laboratory conditions, researchers have shown that efflux pump inhibitors can impair the virulence of several pathogens. Evidence shows that some RND transport proteins participate in the efflux of virulence-related components of bacteria. PAβN is the most efficient inhibitor of the *P. aeruginosa* RND efflux pump and reduces some of the virulence characteristics of this bacterium. PAβN attenuated the virulence of *P. aeruginosa* PAO1 by inhibiting processes related to motility and micronutrient acquisition (i.e., phosphate and iron). Meanwhile, in the PAO1 strain, PAβN stimulated the generation of N-3-oxododecanoyl-homoserine lactone (3OC_12_-HSL) and pyocyanin, which plays an active role in its virulence [96]. At the transcriptional level, PAβN markedly decreased the relative expression of QS cascades, and the QS-regulated type II secretion gene lasB and toxA (exotoxin A). In addition, PAβN diminished the relative expression of pelA (exopolysaccharides) in clinical virulent isolates. PAβN significantly blocked the QS circuit and inhibited the virulence factors expressed by clinical isolates of *P. aeruginosa*, rendering them less virulent (Figure 8) [97]. In another study, NMP and PAβN reduced the production of cholera toxin and the number of toxin co-regulatory trichomes (TCPs). Various concentrations of PAβN or NMP reduced the expression of the *tcpPH* and *toxT* genes which encode essential regulators of *Vibrio cholerae* virulence genes. They appeared to suppress the expression of other virulence genes independently of RND efflux inhibition. Thus, PAβN and NMP may have additional effects on virulence gene expression [98].

Overall, pathogen resistance and pathogenicity are decreased by efflux pump inhibitors. Therefore, they can increase the success of antimicrobial therapy when treating infections while potentially reducing the harmful effects of infections on patients [31]. Efflux inhibition may be an essential method to control bacterial virulence and pathogenesis.

### 4.3. Efflux Pump Inhibitors Reduce the Formation of Bacterial Persister Cells

When it comes to medication therapy, the majority of cells are swiftly eradicated by bactericidal antibiotics. Still, a relatively tiny number of phenotypic variants, known as persister cells, exhibit considerable resistance [99]. The essential difference between persister cells and drug-resistant bacteria in the traditional sense is that the latter result from genetic mutations which prevent the drug from binding to the target in various ways, resulting in the inhibition of the drug’s effect. On the other hand, persister cells represent non-genetic phenotypic variation and have a dormant phenotype: almost all life activities have stopped, so even though the drug can bind to the corresponding target, it does not exert a toxic effect on the microorganism. Hence, persister cells tolerate almost all drugs [100]. 

In persister cells, antibiotic accumulation can be decreased through an increase in efflux, a decrease in cell membrane permeability, or both. Transcriptome sequencing has confirmed that multidrug efflux gene expression is markedly higher in persister cells. Cells with efflux gene upregulation show enhanced persistence, while efflux knockout mutants show an attenuation of persistence, suggesting that efflux pumps play an essential role in bacterial drug resistance. The AcrAB-TolC efflux pump is present in most Gram-negative bacteria. It has a proton-dependent mechanism for releasing hazardous metabolic intermediates and at least nine antibiotics. The lack of AcrB and TolC impairs the persistence of delafloxacin in planktonic stationary phase cultures of *E. coli*. The co-administration of PAβN (an inhibitor of AcrAB-TolC) and delafloxacin resulted in a 40-fold reduction in the survival of stationary phase *E. coli* populations after 7 h of treatment compared with cultures treated with delafloxacin and dimethyl sulphoxide. Targeting the AcrAB-Tol C efflux pump enhanced delafloxacin activity against slow/non-growing *E. coli* during treatment [101]. Pu et al. similarly identified that persisters show higher levels of expression of efflux-related genes. A high level of expression of tolC is essential for promoting persistent cell formation, and persisters combine active efflux and passive dormancy to survive antibiotic attack. They found a significant reduction in persister cells after the addition of efflux pump inhibitors when comparing the use of antibiotics alone with the antibiotics plus PAβN or NMP [99].

Consequently, the combination of efflux pump inhibitors with conventional antibiotics holds promise for the treatment of persistent bacterial disease by reducing intrinsic resistance or acquired multidrug resistance associated with efflux pumps. These compounds assist antibiotics to clear persister cells, thereby ultimately killing the pathogenic microbial population.

### 4.4. Efflux Pump Inhibitors Limit the Acquisition of Bacterial Resistance

The capacity of bacteria to access genes through horizontal transfer mechanisms is a major cause of the spread of drug resistance. Horizontal gene transfer (HGT) is a driver of antimicrobial resistance gene transmission in bacteria. Drug resistance genes for clinically significant antibiotics are typically carried on plasmids, which are circular DNA segments that can be conjugated between several bacterial species. Studies have shown that even when drugs that restrict gene expression exist, bacteria can still express resistance genes [102]. Researchers revealed the critical role of multidrug efflux complexes in maintaining drug-specific resistance through horizontal gene transfer in the presence of bacterial inhibitors [103]. One reason is that the plasmid delivers both the Tet A resistance protein and the plasmid to the receptor. The transferred Tet A protein can pump out enough tetracycline to allow for translation, which produces more Tet A from the plasmid. AcrAB-TolC is essential for the acquisition of tetracycline resistance via horizontal gene transfer, which may ensure that the newly acquired plasmid is able to synthesise TetA resistance by decreasing the tetracycline content of the cells. When we use antibiotics to treat bacterial infections, it is possible that horizontal gene transfer through the digestive tract may lead to the emergence of new drug-resistant strains, resulting in therapeutic failure. Therefore, in addition to inhibiting bacterial growth through antibiotics, reducing the horizontal transfer of antibiotic resistance may be considered. Antibiotic resistance is mainly attributed to the AcrAB-TolC efflux pump, which raises the possibility that blocking the multidrug efflux system is a significant contributing factor. Efflux pump inhibitors might have an indirect effect by preventing the spread of antibiotic resistance among bacteria during therapy, which would stop the creation of new resistant strains [102].

### 4.5. Efflux Pump Inhibitors Improve Mismatch Repair

Most organisms contain DNA mismatch repair (MMR) systems to prevent mutations and preserve genomic stability. MMR fixes mismatches and insertion–deletion loops caused by DNA synthesis, increasing DNA replication’s overall fidelity by up to a thousand times. *MutS* initiates MMR by identifying mismatches in newly replicated DNA [104]. Antibiotic resistance is mainly the result of genetic changes, but bacteria can also resist drug treatment by transiently expressing multidrug efflux pumps. El Meouche et al. focused on transient drug resistance caused by the heterogeneity of AcrAB-TolC efflux pump expression found in various infections. The authors built a two-colour plasmid to record the expression of the *acrAB* and *mutS* promoters in order to study the relationship between *acrAB* and *mutS* expression. Single-cell time-lapse fluorescence microscopy showed that cells with greater levels of *acrAB* expression had much lower levels of *mutS*, making them more susceptible to mutation. Transient antibiotic resistance from increased *acrAB* expression could facilitate spontaneous mutations that lead to permanent antibiotic resistance [105]. This suggests that cells with increased efflux pump expression are more resistant to antibiotics and also have less MMR, making them more susceptible to mutations. We could potentially apply efflux pump inhibitors in combination with antibiotic treatment, which can reduce the expression of efflux pumps and thus increase the probability of MMR to prevent the emergence of drug resistance.

## 5. Summary and Prospects

Efflux pump inhibitors can restore the antibiotic efficacy of clinically important pathogenic microorganisms and can play a vital role in solving antibiotic resistance. The mechanisms of action of efflux pump inhibitors include competitively blocking the binding sites on the efflux pump, reducing drug efflux by competing with antibiotics for the active site of the bacterial efflux pump [44,106], and the gene-level inhibition of exocytosis pump expression or selective inhibition of the expression of genes encoding exocytosis pumps through antisense nucleotides or small-molecule RNA. The expression of *acrAB* in *E. coli* and the expression of *cmeABC* in multidrug-resistant *C. jejuni* have been inhibited using antisense nucleic acids and oligonucleotides, respectively [107].

**Figure 9:**
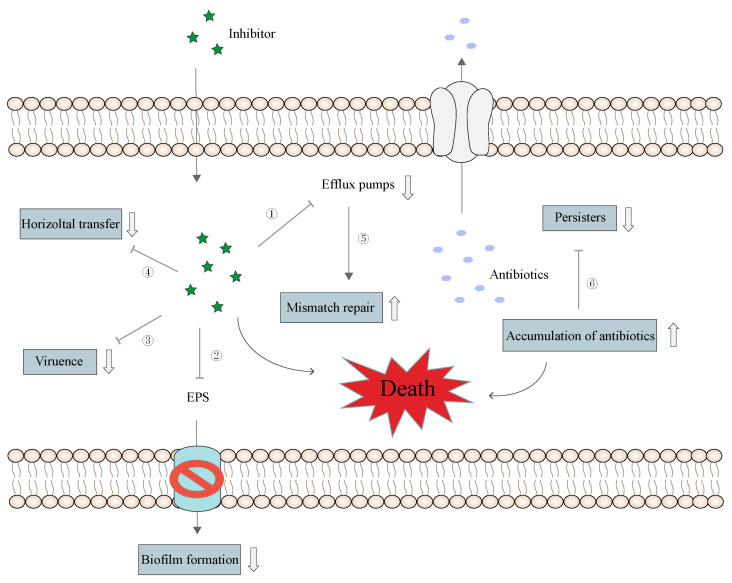
Schematic diagram of the synergistic effects of efflux pump inhibitors and antibiotics. ① Inhibitors promote antibiotic accumulation in cells by inhibiting the multidrug efflux pump function; ② disrupt EPS transport and inhibit biofilm formation; ③ impair bacterial virulence; ④ prevent horizontal transfer of resistance; ⑤ enhance mismatch repair; and ⑥ assist in antibiotic clearance of persister cells.

The inhibitors we have described in this review include natural and synthetic compounds. They have shown promising efficacy in delaying bacterial resistance when used with antibiotics (Figure 9). These inhibitors have multiple benefits such as facilitating gastrointestinal absorption, improving blood–brain barrier penetration, and increasing drug concentration in mammalian cells to enhance the killing of invasive pathogens [93]. Therefore, developing safe and effective efflux pump inhibitors is essential to improve the activity of antibacterial drugs and the current status of the clinical treatment of bacterial infections.

Currently, most efflux pump inhibitors are still in the preclinical phase or in vitro trials. There are numerous challenges when developing novel antibiotics or clinically effective efflux pump inhibitors that can circumvent multiple drug-resistant bacteria. Due to their poor stability, poor selectivity, and severe cytotoxicity to eukaryotic cells, inhibitors have not yet been employed treating bacterial infections [108]. Efflux pump inhibitors as therapeutic agents present several difficulties, but their value and benefits should not be overlooked. They have long been recognized as drugs that can increase the effectiveness of antibiotics against multidrug-resistant bacteria, and there have been numerous studies backed by data demonstrating their effectiveness. Nevertheless, more investigation is required to fully realize the potential of efflux pump inhibitors.

## Figures and Tables

**Table 1:** The mechanisms of drug resistance in common clinical pathogens.

Bacteria	Drug Resistance Mechanism	References
*Staphylococcus aureus*	Reduced affinity for penicillin-binding proteins and the simultaneous production of β-lactamases to destroy the antibacterial activity of the drug.	[8,9,10]
	An eEnhanced efflux effect; the most effective multidrug resistance system is NorA.	
	A decrease in cell membrane permeability affects the energy metabolism of bacteria, reduces the absorption of drugs, and leads to drug resistance.	
*Klebsiella pneumoniae*	The generation of 16S rRNA methylesterase and β-lactamase, the mutation of target genes, the generation of multidrug resistance efflux pumps, and the modification of enzymes and target protection proteins.	[11]
*Pseudomonas aeruginosa*	Metallo-β-lactamase-producing enzymes, target mutations, alterations in membrane pore proteins, and active exocytosis systems.	[12]
*Escherichia coli*	The production of β-lactamase, the inhibition of bacterial DNA topoisomerase, and a reduction in drug uptake and increase in efflux through outer membrane alteration.	[13]
*Campylobacter jejuni*	Alteration of antibiotic targets or their expression, exocytosis (e.g., CmeABC), and the modification or inactivation of antibiotics.	[14]

**Table 2:** Examples of relevant efflux pumps for common clinical pathogens.

Bacteria	Efflux Pump	Family	Efflux Substrate	References
*Staphylococcus aureus*	Nor A	MFS	β-lactams, fluoroquinolones, tetracyclines, fungicides, dyes, quaternary ammonium compounds, and preservatives	[22,23,24]
*Klebsiella pneumoniae*	AcrAB-TolC	RND	β-lactams, macrolides, fluoroquinolones, tetracycline, chloramphenicol, cationic dyes, and detergents	[25]
	OqxAB	RND	Quinolones, chloramphenicol, olaquindox, and tigecycline	
*Pseudomonas aeruginosa*	MexAB-OprM	RND	Quinolones, tetracyclines, macrolides, chloramphenicol, neomycin, and lincomycin, but not the β-lactam imipenem	[6,26]
	MexCD-OprJ		Quinolones, tetracyclines, macrolides, neomycin, lincomycin, chloramphenicol, flomoxef, and meropenem	
*Escherichia coli*	AcrAB-TolC	RND	Fluoroquinolones, tetracyclines, macrolides, rifampin, linezolid, oxazolidinone, novobiocin, clindamycin, and chloramphenicol	[27]
*Campylobacter jejuni*	CmeABC	RND	Macrolides, fluoroquinolones, tetracyclines, rifampin, toxic chemicals, and bile acids	[28,29,30]

**Table 3:** Types of efflux pump inhibitors and their sources.

Source	Inhibitor Type	Representative Drugs	Suppressed Efflux Pumps	References
Plants	Alkaloids	Reserpine	NorA	[32]
		Berberine	MexXY-OprM and NorA	[33]
		Piperine	NorA, MdeA, and Rv1258c	[34,35,36]
	Flavonoids	Astragalusin, kaempferol, silymarin	NorA	[32,37,38]
		Chalcone	NorA and MepA	[39]
		Lignocaine	MsrA	[40]
	Phenolic metabolites	5′-MHC	NorA	[41]
	Fermentation products	EA-371α and EA-371δ	MexAB-OprM	[15]
	Gallic acid	Epigallocatechin gallate	TetK, MexAB-OprM, and CmeABC	[42,43]
Chemical synthesis	Pyranopyridine	MBX2319	AcrAB-TolC	[44]
	Peptides	Phe-Arg-β-naphthylamide	MexAB-OprM, MexEF-OprN, and MexCD-OprJ	[45]
	Phenothiazines	Chlorpromazine	AcrB	[46]
	Aryl piperazine derivatives	NMP	AcrAB and AcrEF	[47]
	Pyridopyrimidine derivatives	D13-9001	AcrB and MexB	[48]

## Data Availability

Not applicable.
